# A meta-analytic approach on Vortioxetine and acute depression

**DOI:** 10.1192/j.eurpsy.2025.2089

**Published:** 2025-08-26

**Authors:** I. Berardelli, E. Rogante, F. Formica, R. Iannazzo, A. V. Mammoliti, R. Riccioni, S. Veizi, R. S. McIntyre, M. Pompili

**Affiliations:** 1 Department of Neurosciences, Mental Health and Sensory Organs, Faculty of Medicine and Psychology, Suicide Prevention Centre, Sant’Andrea Hospital, Sapienza University of Rome, Via di Grottarossa, 1035, 00189; 2 Department of Human Neurosciences, Sapienza University of Rome; 3 Psychiatry Residency Training Program, Faculty of Medicine and Psychology, Sapienza University of Rome, Psychiatry Unit, Sant’Andrea Hospital,00185, Rome, Italy; 4 Department of Psychiatry, Canada Department of Pharmacology, University of Toronto, Toronto, Canada

## Abstract

**Introduction:**

Major Depressive Disorder (MDD) is a chronic, recurrent illness characterized by various combinations of signs and symptoms, severity levels, and loss of functions. Among the available pharmacological treatments for MDD, Vortioxetine, a serotonin transporter inhibitor (SERT), has been widely used for its multimodal action on serotonin neurotransmission, which produces important changes also on glutamate, GABA, norepinephrine, acetylcholine, and dopamine.

**Objectives:**

The aim of this systematic review and meta-analysis is to evaluate the acute efficacy of Vortioxetine across multiple dosing to evaluate if there is a dose response effect and as well is there a dose response issue with respect to side effects.

**Methods:**

According to PRISMA guidelines we systematically searched 3 major electronic databases (PubMed/MEDLINE, PsycINFO, and Cochrane Central Register of Controlled Trials) for Randomized Controlled Trial (RCT) studies published between January 2013 and April 2024. The RCTs evaluate the efficacy of Vortioxetine on acute depression, compared with placebo or other antidepressants, through improvements in depressive symptoms based on MADRS or HAM-D scores. Twenty-four studies were included in the analysis.

**Results:**

In general, Vortioxetine significantly improved depression severity, anxiety symptoms, cognitive function, with high response and remission rates. It was also well tolerated with a relatively low occurrence of severe or serious treatment emergent adverse events (TEAEs), with nausea as the most frequent adverse event, and/or discontinuation due to intolerability. Observing the results of the meta-analysis, the effect was significant for both Vortioxetine 10 and 20 mg with a greater effect-size for vortioxetine 20 mg. These different results could explain a better clinical efficacy of Vortioxetine 20 mg than Vortioxetine 10 mg for acute symptoms of depression.

**Conclusions:**

In conclusion, the results of the present study underlined the efficacy and safety of Vortioxetine and pointed out that the optimal dosage of Vortioxetine for the treatment of acute symptoms of depression varies between 10 and 20 mg with 20 mg for a better management of acute depression. When choosing initial therapy for MDD in clinical practice, it is important to consider not only drug efficacy but also patient preferences such as AEs and possible adherence. Thus, Vortioxetine should be consider efficacious as a first, and second line therapy.

**Disclosure of Interest:**

None Declared

